# Early Neurodevelopmental Outcomes after Previable Preterm Prelabour Rupture of Membranes (pPPROM)

**DOI:** 10.1155/2022/3428841

**Published:** 2022-09-20

**Authors:** Christy L. Pylypjuk, Katarina Nikel, Chelsea Day, Ladonna Majeau, Adelicia Yu, Yasmine ElSalakawy, M. Florencia Ricci

**Affiliations:** ^1^Department of Obstetrics, Gynaecology and Reproductive Sciences, University of Manitoba, Winnipeg, Manitoba, Canada; ^2^Children's Hospital Research Institute of Manitoba, Winnipeg, Manitoba, Canada; ^3^Department of Obstetrics, Gynecology and Reproductive Sciences, University of Saskatchewan, Saskatoon, Saskatchewan, Canada; ^4^Department of Pediatrics and Child Health, University of Manitoba, Winnipeg, Canada

## Abstract

**Objective:**

To describe the early neurodevelopmental outcomes following fetal exposure to previable preterm prelabour rupture of membranes (pPPROM).

**Methods:**

This was a secondary analysis of a subgroup of neonates born following pPPROM from a retrospective cohort study (2009–2015). Surviving infants who underwent standardized neurodevelopmental evaluation at 18–24 months corrected age (CA) between 2017 and 2019 were eligible for inclusion. Data abstracted from hospital charts were linked to prospectively collected developmental outcomes stored in an electronic database from a regional neonatal follow-up clinic. The primary outcome was Bayley-III composite scores (compared to the population mean 100, standard deviation (SD) 15). Secondary outcomes included presence of cerebral palsy, vision loss, hearing impairment, and requirement of rehabilitation therapy. Descriptive statistics were used to present results.

**Results:**

25.7% (19/74) of neonates born after pPPROM survived to hospital discharge, but only 21.6% (16/74) survived to 18–24 months CA. Of these, 9 infants were eligible for follow-up at the regional clinic and 7 had developmental outcomes stored in the electronic database. Infants exposed to pPPROM exhibited Bayley-III scores more than 1 SD below the population mean across all three domains: cognitive 84.9 (SD 12.2); motor 82.3 (SD 11.5); and language 66.4 (SD 18.9). There were particular deficiencies in language development with 71% (5/7) scoring more than 2 SDs below the population mean. There were no cases of cerebral palsy.

**Conclusions:**

Only 1 in 5 infants born following expectantly managed pPPROM survived to 18–24 months CA. These infants born after pPPROM had significantly lower Bayley-III scores and particular deficiencies in language development. Better understanding of early neurodevelopmental challenges following pPPROM will help refine counselling of families contemplating expectant management and provide insights into the postnatal educational resources required to improve long-term developmental outcomes for these children.

## 1. Introduction

Previable preterm prelabour rupture of membranes (pPPROM) is a rare complication of pregnancy, affecting ∼1–8 per 1000 births [1, 2]. Preterm delivery and related neonatal complications of prematurity, abruption, stillbirth, intrauterine infection, and associated infectious morbidity are some of the adverse outcomes related to preterm prelabour membrane rupture [2–4]. With membrane rupture occurring prior to viability, there are additional concerns about pulmonary hypoplasia due to low amniotic fluid volumes at the time of critical lung development [1–3, 5–7]. The prognosis after pPPROM is generally guarded, with many families choosing not to continue the pregnancy [8, 9]. To date, much of the existing literature about pPPROM has focused on obstetrical outcomes and neonatal survival to hospital discharge [5–7]. Perinatal mortality following pPPROM has been reported as high as 95% [2]. Low amniotic fluid is associated with adverse outcomes, but the association between amniotic fluid volumes and developmental outcomes is currently unknown [10–12]. For instance, there was a higher frequency of stillbirth and previable delivery with complete anhydramnios postrupture [10]. In another study, a minimum single deepest vertical pocket of at least 1 centimeter or more was associated with longer latency and greater neonatal survival [11]. There was also a higher risk of caesarean section, postpartum hemorrhage, and maternal sepsis after pPPROM [7, 10]. However, less is known about the long-term, postnatal outcomes of neonates following pPPROM.

Prematurity is a known risk factor for long-term disability [12–14]. At 2 years of age, there is a higher incidence of motor delay in children born preterm with almost one-third having Bayley-III motor scores below 85 [15]. Very preterm birth—particularly in the setting of intrauterine infection—is strongly associated with severe neurodevelopmental impairment, including cerebral palsy [13, 15–20]. However, reports on the frequency of chorioamnionitis in the setting of PPROM are highly variable and range between 40% and 93% [19, 21]. There are also challenges with ascertaining whether the intrauterine infection precedes membrane rupture or follows as a consequence of ascending infection through an opening in the membranes. In our original study with over 113 cases of pPPROM, there were higher rates of chorioamnionitis in the expectant management group versus those undergoing termination, suggesting that not all cases of preterm membrane rupture necessarily occur as a consequence of infection [10]. While latency does seem to be associated with risk of chorioamnionitis, one study did not find any obvious difference in prevalence of severe impairment by categorical latency duration of greater that 3 weeks (versus <3 weeks) [17].

With advances in perinatal medicine, neonatal survival from pPPROM is possible, yet there is little information to guide counselling of families about the long-term risks of this pregnancy complication. The goal of this study is to determine the neurodevelopmental outcomes in early childhood following survival from pPPROM. Better understanding of the long-term outcomes of these patients could help refine counselling of parents and aide in decision-making about pregnancy management. For survivors, enhanced knowledge of the specific impact on neurodevelopment would allow for targeted interventions and educational supports sooner, in order to improve outcomes for this high-risk pediatric group.

## 2. Materials and Methods

This was a secondary analysis of a subset of infants born between January 1, 2009, and December 31, 2015, following pPPROM from a previously published retrospective cohort [10]. Those infants presenting to a regional neonatal follow-up clinic with stored developmental outcomes at 18–24 months CA (June 1, 2011 to December 31, 2017) were eligible for inclusion. The neonatal follow-up clinic serves as one of the two referral sites for a regional population of over 1.3 million and a territory spanning urban, rural, and northern/remote communities. As per local practice standards, all neonates born preterm and admitted to the neonatal intensive care unit in the region are offered postnatal follow-up at the neonatal follow-up clinic associated with each respective maternity hospital. The neonatal follow-up clinic is staffed by developmental pediatricians, neonatologists, occupational therapists, physiotherapists, audiologists, and nurses. Assessments of neurodevelopment for this population are performed in the standard manner at 6–12 months CA and 18–24 months CA, including evaluation of hearing, vision, cerebral palsy, and with the Bayley Scales of Infant and Toddler Development-Third Edition(Bayley-III) [22]. Research ethics approval was obtained from the University of Manitoba's Human Research Ethics Board. Individual patient consent (or ascent) and parental consent is obtained in the standard manner for inclusion of developmental outcomes data in the clinical database, and site approval for this project was obtained from the Specialized Services Centre for Children and Youth in Manitoba.

From the original cohort of pregnant patients diagnosed with pPPROM prior to 24 + 0 weeks of gestation during the study period, those neonates surviving to hospital discharge were assessed for possible inclusion [10]. To start, all pregnancies complicated by PPROM had been identified using the International Classification of Disease–Ninth Revision (ICD-9) coding by hospital discharge abstracts [10]. From those cases coded as PPROM, the subgroup of those with previable membrane rupture prior to 24 + 0 weeks of gestation were confirmed using manual hand-searches of the hospital records, and diagnosis of membrane rupture required documented presence of pooling and ferning on speculum examination. Pregnancies with planned postnatal palliation, iatrogenic membrane rupture, rescue cerclage within 14 days, congenital anomalies, multiples, prelabour rupture of membranes occurring at gestational ages after viability, and cases with latency less than 24 hours were excluded. Manual searches of the paper-based hospital charts were also used to collate information about basic demographics, obstetrical history, fetal ultrasound results, birth events, and neonatal course in hospital [10]. Cases were then linked to prospectively collected neurodevelopmental outcome measures at 18–24 months CA and stored in the electronic database at the neonatal follow-up clinic. The primary outcome was Bayley-III cognitive, language, and motor composite scores. Secondary outcomes included: (i) presence of cerebral palsy (determined by reports from neurology and/or by the neonatologist/developmental pediatrician in the follow-up clinic); (ii) and presence of vision loss; (iii) hearing impairment (defined by hearing test results). Need for hearing aids or cochlear implants were obtained from audiology reports); and (iv) requirement of allied health supports (occupational therapist, physical therapist, and speech language pathologist).

Descriptive statistics were used to present the results. Continuous variables were presented as means with standard deviations (SD) if normally distributed or as medians with interquartile ranges (IQRs) if nonparametrically distributed. Dichotomous and categorical variables were described as proportions (in %). Additionally, Bayley-III scores were compared against the known population means and reference ranges: a score of 100 represents the population mean, and scores of 85 and 70 represent −1 and −2 standard deviations below the mean, respectively [22]. Statistical analysis was performed using Stata v.14.2 (Stata Corp, College Station, TX, USA) software.

## 3. Results

**Figure 1:**
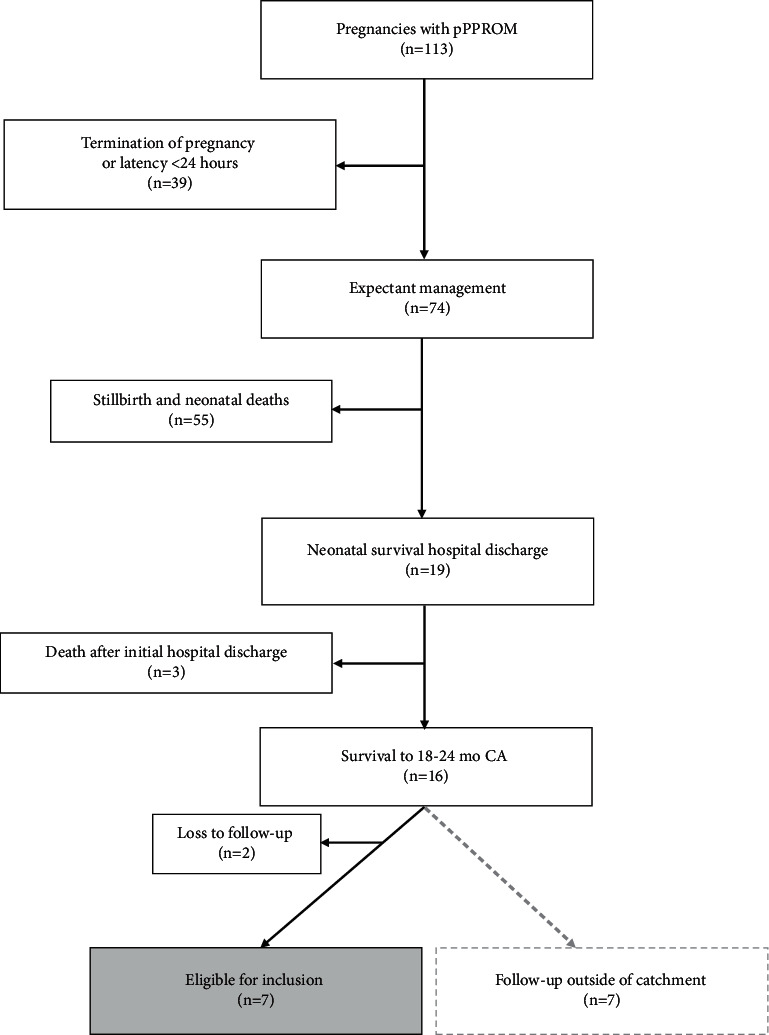
Flowchart of patients included in the study. pPPROM, previable preterm prelabour rupture of membranes; CA, corrected age.

From 113 pregnancies complicated by pPPROM between 2009 and 2015, 74 opted for expectant management and achieved latency ≥24 hours (Figure 1). In both maternity hospitals (sites A and B), approximately 1 in 4 neonates survived to hospital discharge (25.6%), but only 21.6% (16/74) survived to 18–24 months CA. Of these 16 survivors, 9 infants were within the referral catchment of the study site for this project. After excluding those without stored developmental outcomes at 18–24 months, there remained 7 infants who attended neurodevelopmental follow-up assessments between 2011 and 2017 and were included in the final analysis (Figure 1).

85.7% (6/7) of neonates were born to multiparous patients (Table 1). The median maternal age was 25 years (IQR 24–38), and most resided in an urban location (71.4%; 5/7). Almost half (42.9%; 3/7) of these pregnant patients had a past obstetrical history significant for at least 1 prior therapeutic or spontaneous abortion. 57.1% (4/7) of these pregnancies had also been complicated by antepartum hemorrhage. Median gestational age at rupture of membranes was 21 weeks (IQR 19 + 2 to 22 + 5). Oligohydramnios at first ultrasound postmembrane rupture was reported in 57.1% (4/7) of cases. Most neonates (85.7%; 6/7) had received at least 1 course of antenatal corticosteroids prior to birth, and there was one case with 2 full courses of antenatal corticosteroids administered prior to birth. Median gestational age at delivery was 25 weeks (IQR 24 + 1 to 28 + 3). 57.1% (4/7) of births occurred via caesarean section, although the indications were varied: 2 were repeat caesarean sections performed because of a prior uterine scar, 1 for malpresentation, and another for fetal distress. There were no cases of chorioamnionitis amongst those eligible for inclusion in this study. Over half of survivors with stored neurodevelopmental outcomes were male (57.1%; 4/7). The median birthweight was 865.5 grams (IQR 768–1347; Table 1). Average length of stay in the NICU was 129.5 days (SD 81.6). Aside from maternal age and birthweight, there were no obvious significant differences in demographics or birth events between those with and without stored developmental outcomes, although the numbers were small. The two neonates lost to follow-up were born to slightly older mothers (median 29.5 years (IQR 24–35.5)) and had higher median birthweights (1153.5 grams (IQR 769.5–1989)).

Bayley-III scores following pPPROM were lower than the population mean across all domains. The average Bayley-III score was 84.9 (SD 12.2) for cognitive and 82.3 (SD 11.5) for motor (Table 2). Bayley-III language scores were most significantly impacted, with an average score of 66.4 (SD 18.9). 85.7% (6/7) of infants had language scores at least 1 standard deviation below the population mean (<85), and 71.4% (5/7) had scores 2 or more standard deviations below (<70). Comparatively, 57.1% (4/7) of infants had motor and cognitive scores 1 standard deviation below the population mean, and none had scores less than 2 standard deviations below in either domain. One child was reported to have vision impairment (14.3%; 1/7), and one child experienced hearing impairment (14.3%; 1/7). There were no reported diagnoses of cerebral palsy in this cohort. Only two children (28.6%; 2/7) were reported to receive additional allied health services at the time of the neurodevelopmental assessment, which comprised occupational therapy, physical therapy, and speech language pathology.

## 4. Discussion

pPPROM is an uncommon but serious complication of pregnancy with high morbidity and mortality. Because the majority of fetuses/neonates exposed to pPPROM do not survive beyond the early neonatal period, less is known about long-term outcomes of this high-risk group. By leveraging an existing 6-year birth cohort of pregnancies complicated by pPPROM, we were able to evaluate the neurodevelopmental outcomes of a subgroup of those with follow-up scheduled in one of our two regional neonatal follow-up clinics [10]. The rate of neonatal survival to hospital discharge amongst those opting for expectant management of our original cohort (25.6%) is comparable to other published studies of pPPROM; however, it was interesting that only 1 in 5 of infants had survived to 18–24 months CA [3, 5–7]. Few other studies have evaluated long-term survival after pPPROM. With only half of survivors' eligible for planned postnatal follow-up at the study site, our project is small but still representative of the larger population from which it was sampled.

In 18–24-month-old survivors of pPPROM, Bayley-III scales were low across all domains and particularly within language development. Even compared to a large national network of pediatric follow-up clinics reporting outcomes for all-cause prematurity, Bayley-III scales in our pPPROM cohort were lower [23]. From that network data, over 100 infants born between 24 and 25 weeks' gestation achieved Bayley-III scores closer to 95, 94, and 89 for cognitive, motor, and language domains, respectively. Only 41% of 25-week-old infants had language scores 1 standard deviation below the population mean, and 14% had scores that were 2 standard deviations below, compared to 85.7% (6/7) and 71.4% (5/7), respectively, amongst our subgroup of pPPROM survivors. It should also be noted that our single-clinic follow-up rate of 77.8% is also similar to the follow-up rates reported by participating Canadian Neonatal Follow-Up Network sites, which range from 63 to 76% [23]. To our knowledge, specific deficiencies in language development post-pPPROM have not been described before.

There is literature to link language delay—either primarily or secondarily—to overall cognitive delays [24]. However, amongst survivors of pPPROM in our analysis, the deficiencies in language were more significant than those in the cognitive domain. We postulate whether the intrauterine environment associated with pPPROM uniquely predisposes offspring to specific vulnerability in language development and beyond that seen in other domains. Identification of language delay in children exposed to pPPROM could allow us to implement earlier interventions to improve long-term outcomes. Studies have shown that increased adult and parental vocalizations in the NICU improve language scores at 18 months of age [25, 26]. Use of mother-infant transaction programs in the NICU has also been shown to increase scores of communication development at 6 months, which is known to be associated with improved language abilities later in life [27]. Initiation of early storybook reading in children less than one year old also promotes language and communication skills [28]. Community-based early interventions have also been shown to improve communicative interactions, vocabulary, and overall language skills in children under 3 years old with developmental delay [29]. Educating parents on language-modelling strategies, focused stimulation, and use of music and books to improve language skills in their child increasedthe confidence level and vocabulary skills in children after 6 weeks, and parents also reported increased engagement with their children [29]. Our study would suggest that established interventions to improve language development should already be undertaken when a baby born following pPPROM is admitted into the NICU [25, 26, 28, 29]. Interventions such as parental book reading, increased maternal/parental speech, or multisensory environments in the NICU have shown to be beneficial for development of very preterm infants [25, 30, 31]. Interventions that focus on the parent-infant relationship and infant development were also found to be the most beneficial in the short- and medium-term development of the child [32]. Only 2 children in our cohort had ongoing support from allied health services (including speech language pathology), which suggests that there may be an underappreciation of the specific challenges in language development for survivors of pPPROM. Another possibility is that, in very young children, the absence of significant motor or cognitive delays might “mask” significant language deficiencies: pediatricians and general practitioners caring for children born after pPPROM should be made aware of this potential relationship earlier to allow sufficient lead-time to improve outcomes. The majority of our families resided in an urban location, so the impact of rural/remote residency on access to these educational resources would also need to be considered. These early interventions are especially important as we know that language delay is associated with poor educational outcomes later in childhood [24]. Ultimately, earlier interventions to improve neurodevelopmental outcomes could redirect a child's entire educational trajectory as there is evidence that (a) early school performance is predictive of school outcomes in later grades (including high school graduation) and (b) poor grade-school performance relates to increased risks of chronic medical conditions, mental illness, and poorer overall health later in adulthood [33–37]. Because greater academic achievement is ultimately linked to better health outcomes later in life, the need to provide early educational supports—even in the immediate postnatal period—is acute both medically and societally [38].

While the linkage between prematurity and development of cerebral palsy is well established, the coexistence of intrauterine infection or inflammation has been show to further heighten this risk [39, 40]. Several studies have shown that chorioamnionitis in pregnant patients with PPROM is predictive of severe neonatal morbidity and perinatal death [41]. Based on other Canadian network data, we would expect approximately 7% of preterm infants born at the same gestational age range of our pPPROM subgroup to be diagnosed with cerebral palsy [42]. We were surprised that there were no cases of cerebral palsy diagnosed in our cohort. While our small sample size may impact the incidence of cerebral palsy, another possible explanation is that none of the infants with neurodevelopmental follow-up had pregnancies complicated by chorioamnionitis. The lack of intrauterine infection amongst survivors to 18–24 months was particularly interesting given that the rate of chorioamnionitis in the original cohort was 58.4% and was more common amongst those opting for expectant management versus those undergoing termination for pPPROM (8%) [10]. Could it be possible that cerebral palsy is underrepresented in neurodevelopmental follow-up studies of pPPROM because those that survive long-term are less likely to have been born after chorioamnionitis? We were underpowered to further evaluate this relationship, but consideration of this immortal time bias does warrant consideration in future studies.

Preterm infants are also known to be at higher risk of vision loss and hearing impairment. Approximately 6% of children born preterm in Canada and admitted to NICU are diagnosed with hearing impairment and only 1% with vision impairment [42]; however, there was only 1 diagnosis of each in our pPPROM cohort. While gestational age at birth is a well-known predictor of developmental outcomes, less is known about other covariates that specifically impact the risk of hearing loss and vision impairment. Historically, frequent and prolonged use of gentamycin in children was a significant risk factor of hearing loss; however, gentamycin is typically avoided in modern perinatal medicine because of this concern [43]. There is also the established relationship between retinopathy of prematurity and prolonged exposure to high-concentration supplemental oxygen—a practice that is also currently avoided by neonatologists [44]. With changes to antibiotic choice and improved stewardship overall, advancements in neonatal resuscitation practices and avoidance of pure oxygen supplementation, and aggressive screening for/treatment of retinopathy of prematurity and hearing loss, it is possible that these complications are becoming less frequent in the general preterm population overall. Other advances in obstetrical care that improve outcomes after prematurity may also influence longer-term outcomes after pPPROM. For instance, use of antenatal corticosteroids has been found to increase survival to hospital discharge in neonates exposed to pPPROM [45]. Use of magnesium sulfate has also been shown to have neuroprotective effects on preterm infants and reduce the risk of cerebral palsy [46, 47]. It remains largely unknown how these exposures (chorioamnionitis, antenatal corticosteroids, magnesium sulfate, or amniotic fluid volumes) influence long-term neurodevelopmental outcomes after pPPROM, and our sample size was too small to evaluate these specific relationships.

This is one of a few studies that have explored the neurodevelopmental outcomes of children exposed to pPPROM and specifically report a possible relationship between pPPROM and language delay. The results of this study draw awareness to the specific developmental vulnerabilities that may be unique to children born after pPPROM and that the need for intervention begins early in the postnatal period. These findings also highlight the need for improved access to more targeted developmental supports and early childhood educational resources for families of children with pPPROM. A main limitation of this study is the small sample size, which precluded our ability to evaluate other potential covariates. While this subgroup of pPPROM survivors with stored neurodevelopmental outcomes was drawn from the larger regional cohort and the results should be reflective of the baseline population, the possibility of selection and/or reporting bias persists and warrants larger multicenter studies in the future. Another limitation of this study was the restriction of the study period to births up to and including 2015 because of a local practice change in the definition of viability from 24 weeks to 23 weeks after 2015: the impact of pPPROM in modifying the neurodevelopment of neonates born at periviability is unknown. Information about socioeconomic status and other potential confounders were not available in our data set, but it should be considered in future studies. This study highlights the need for larger, multicentre prospective studies to better evaluate even longer-term developmental outcomes in children surviving from this infrequent complication of pregnancy. As neonatal medicine improves—and consequently, the survival from pPPROM—the need for better access to targeted educational supports for improving childhood outcomes will only increase.

## 5. Conclusions

Through this small observational study, we have described the neurodevelopmental deficiencies (particularly in language development) amongst survivors of pPPROM. Identification of pPPROM as a potential additional risk factor of language delay and neurodevelopmental vulnerability will improve counselling and opportunities for targeted interventions earlier. Future studies are still needed to comprehensively evaluate long-term neurodevelopment and health outcomes in this uncommon but high-risk population.

## Figures and Tables

**Table 1:** Pregnancy characteristics of survivors of pPPROM attending neonatal clinic follow-up at 18–24 months CA.

Characteristics	Infants with neurodevelopmental outcomes at 18–24 months CA (*n* = 7)
Maternal age, median (IQR)	25 (24, 38)
Gravity, median (IQR)	3 (3, 4)
Parity, median (IQR)	1 (1, 2)
Multiparous, % (*n*/*N*)	85.7% (6/7)
Urban residence, % (*n*/*N*)	71.4% (5/7)
Prior preterm birth, *n*	0
Prior abortion, % (*n*/*N*)	42.9% (3/7)
Prior IUFD, % (*n*/*N*)	14.3% (1/7)
GA at rupture of membranes, median (IQR)	21 + 0 (19 + 2 to 22 + 5)
Oligohydramnios, % (*n*/*N*)	57.1% (4/7)
Antepartum hemorrhage, % (*n*/*N*)	57.1% (4/7)
Antenatal corticosteroids,% (*n*/*N*)	85.7% (6/7)
Mean GA at delivery	25 (24 + 1 to 28 + 3)
Delivery via caesarean section, % (*n*/*N*)	57.1% (4/7)
Birthweight (in grams), mean (SD)	865.5 (768–1347)
Male sex, % (*n*/*N*)	57.1% (4/7)
Length of stay in NICU (in days), mean (SD)	129.5 (81.6)

*Note*. pPPROM, previable preterm premature rupture of membranes; IQR, interquartile range; IUFD, intrauterine fetal demise; GA, gestational age; NICU, neonatal intensive care unit; SD, standard deviation.

**Table 2:** Neurodevelopmental outcomes at 18–24 months CA amongst survivors of pPPROM.

Neurodevelopmental outcomes	Infants with follow-up at 18–24 months CA (*n* = 7)
Bayley-III motor, mean (SD)	82.3 (SD 11.5)
Bayley-III cognitive, mean (SD)	84.9 (SD 12.2)
Bayley-III language, mean (SD)	66.4 (SD 18.9)
Hearing impairment, % (*n*/*N*)	14.3% (1/7)
Vision loss, % (*n*/*N*)	14.3% (1/7)
Cerebral palsy, *n*	0
Allied health supports, % (*n*/*N*)	28.6% (2/7)

*Note*. CA, corrected age; SD, standard deviation.

## Data Availability

The data used to support the findings of this study may be available from the corresponding author upon reasonable request.
